# Can Outreach Training and Supportive Supervision Improve Competency in Malaria Service Delivery? An Evaluation in Cameroon, Ghana, Niger, and Zambia

**DOI:** 10.4269/ajtmh.23-0150

**Published:** 2023-12-04

**Authors:** Ruth A. Ashton, Matt Worges, Albert Zeh Meka, Paul Yikpotey, Irenee Domkam Kammogne, Pascalina Chanda-Kapata, Caroline Vanderick, Elizabeth Streat, Joshua Yukich

**Affiliations:** ^1^ School of Public Health and Tropical Medicine, Tulane University, New Orleans, Louisiana;; ^2^ Tropical Health, New Orleans, Louisiana;; ^3^ Tropical Health, Yaoundé, Cameroon;; ^4^ Tropical Health, Accra, Ghana;; ^5^ Tropical Health, Niamey, Niger;; ^6^ Tropical Health, Lusaka, Zambia;; ^7^ Tropical Health, Barcelona, Spain;; ^8^ Tropical Health, Maputo, Mozambique

## Abstract

Outreach Training and Supportive Supervision (OTSS) of malaria services at health facilities has been adopted by numerous malaria-endemic countries. The OTSS model is characterized by a hands-on method to enhance national guidelines and supervision tools, train supervisors, and perform supervision visits. An independent evaluation was conducted to evaluate the effectiveness of OTSS on health worker competence in the clinical management of malaria, parasitological diagnosis, and prevention of malaria in pregnancy. From 2018 to 2021, health facilities in Cameroon, Ghana, Niger, and Zambia received OTSS visits during which health workers were observed directly during patient consultations, and supervisors completed standardized checklists to assess their performance. Mixed-effects logistic regression models were developed to assess the impact of increasing OTSS visit number on a set of eight program-generated outcome indicators, including overall competency and requesting a confirmatory malaria test appropriately. Seven of eight outcome indicators showed evidence of beneficial effects of increased OTSS visits. Odds of health workers reaching competency thresholds for the malaria-in-pregnancy checklist increased by more than four times for each additional OTSS visit (odds ratio [OR], 4.62; 95% CI, 3.62–5.88). Each additional OTSS visit was associated with almost four times the odds of the health worker foregoing antimalarial prescriptions for patients who tested negative for malaria (OR, 3.80; 95% CI, 2.35–6.16). This evaluation provides evidence that successive OTSS visits result in meaningful improvements in indicators linked to quality case management of patients attending facilities for malaria diagnosis and treatment, as well as quality malaria prevention services received by women attending antenatal services.

## INTRODUCTION

Quality case management is critical to reducing the burden of malaria, including ensuring that patients receive a parasitological diagnosis and, when confirmed to have malaria, receiving prompt and appropriate treatment. With health systems in malaria-endemic areas continuing to face pressures from resource constraints (human, financial, and commodities), optimizing the quality of care received by patients seeking care within the public health sector is both of high importance and inherently challenging in a dynamic environment.

More than 15 years after the widespread rollout of rapid diagnostic tests (RDTs) for parasitological confirmation of malaria and the introduction of artemisinin-based combination treatment (ACT) for uncomplicated malaria, there continue to be gaps in the quality of diagnosis and treatment of malaria.^1^ Intermittent preventive treatment in pregnancy (IPTp) with sulfadoxine–pyrimethamine (SP), a safe and effective method to protect mother and child from malaria, continues to have low coverage. Among 33 African countries with IPTp policies and moderate to high transmission, only 55% of eligible pregnant women during 2021 were estimated to have received the first dose of IPTp, and only 33% received the third IPTp dose.^2^ Despite national guidelines emphasizing the importance of diagnosis, treatment, and IPTp, ensuring quality of service within health facilities is a critical challenge.

A range of strategies has been tested to improve quality of malaria case management, with available evidence and estimated effects compiled in the Health Care Provider Performance Review in 2018.^3^ Although comparing the effect size of interventions among studies is challenging because of the diverse range of interventions tested and methodological heterogeneity, training and supervision interventions are one of the most commonly explored interventions, and they have a moderate effect size.^3^ An area identified for further research included identification of the attributes of training and supervision that are associated with effectiveness to refine these tools and enhance their effectiveness further. Supportive supervision as an intervention to improve quality of care has been widely adopted and is often considered a core health system strengthening approach,^4^ but has varying interpretations, and implementation can be irregular and have low coverage.^5^

Outreach Training and Supportive Supervision (OTSS) for malaria care improvement has been adopted by a number of low- and middle-income countries as part of a continuous improvement process. Supportive supervision approaches emphasize building relationships between health facility staff and supervisors who model their approach to performance improvement after a coach or mentor.^6^ Emphasis is also placed on using supervisory visit-generated data to monitor performance and to solve problems jointly under a paradigm that avoids finding fault with or enforcing punitive actions on specific individuals.^6^ OTSS activities can be considered a more hands-on method than existing routine supervision approaches, encompassing revision of national guidelines and supervision tools, consistent supervisor training and debriefing, and resources for supervisor travel. The pathways to change from outputs to outcomes are dependent on a combination of mutually reinforcing activities underpinned by dissemination of national guidelines (e.g., malaria case management, drug-based malaria prevention, malaria in pregnancy [MIP]) and the presence of trained health workers across health system levels (public, private for profit, private not for profit, and community). The OTSS approach assumes that for patients to receive quality services, key outputs must be achieved: standardized checklists, a pool of highly qualified supervisors, the capacity to analyze and interpret the data generated, and the capacity to address areas of concern.

Previous research on the initial evaluation of the malaria-specific OTSS model (the Improving Malaria Diagnostics [IMaD] Project, 2008–2012), which used a multistep checklist to track performance, found modest but significant improvements in malaria microscopy and RDT performance in Zambia (∼14% increases), as well as improvements in fever case management practices and prescriber adherence to negative malaria test results (∼7% increases) from the first to the fourth OTSS visit among 88 laboratory and 64 clinical settings.^7^ Assessed laboratories were also found to have significant declines in stock outages of microscopy reagents/consumables, with significant increases in instituting the use of microscopy positive controls and conducting malaria parasite quantification.^7^

After the second incarnation of the OTSS model for malaria (the MalariaCare Project, 2012–2017), a seven-country joint analysis that covered 1,037 health facilities found modest improvements in blood slide preparation and interpretation after two to three supervisory visits.^8^ Overall malaria microscopy performance assessed by a 30-step checklist found the mean score at baseline was 85.7%, and predicted an increase of 3.6% (*P* <0.001) in facility scores after three visits.^8^ Assessment of change in RDT performance across eight countries covering 3,603 health facilities estimated that performance improved modestly, by 5.3%, from the first to the second visit (*P* <0.001), and improved only by 0.6% (*P* = 0.10) between the second and third visits; however, average baseline scores were relatively high at 85%.^9^ The same program provided supervisory visits to 3,563 health facilities across eight countries and, using a 25-point checklist for direct observation of patient consultations, found a 6.3% increase on overall clinical performance (*P* <0.001) related to febrile case management from the first to the third visit. After three visits, the analysis noted an increased percentage of health workers checking for factors associated with malaria-related morbidity and mortality (i.e., signs of severe malaria, pregnancy, and anemia).^10^

A set of best practices were identified after the MalariaCare project, which implemented activities across 17 sub-Saharan African countries, including the development of performance evaluation tools that emphasize opportunities and prioritize time for mentorship in addition to data collection, the use of competency criteria in the selection of supervisors coupled with ongoing skills assessment of those selected, a dynamic health facility selection scheme for enrollment into the OTSS program that assesses the intensity of and overall need for support continually, and a system that promotes the analysis of OTSS visit–generated data for decision making.^11^

The current independent evaluation aimed to generate evidence on the effectiveness of the current malaria OTSS approach (Impact Malaria Project, 2018–2022) on malaria case management performance, RDT and microscopy performance, and on the use of IPTp during routine antenatal care (ANC) consultations in Cameroon, Ghana, Niger, and Zambia, specifically if receipt of an increased number of OTSS visits to a facility resulted in improved performance as assessed through OTSS checklists. The Impact Malaria Project introduced the OTSS+ approach, which is described as a new approach of supportive supervision at the facility level that uses standard automated checklists centered on continuous improvement of the competencies of health providers in malaria diagnosis and treatment.^12^ Specifically, this approach streamlines existing checklists used for health worker observation; includes a new module to assess provider competency in prevention and treatment of MIP during ANC; uses a digital software platform to process data instantly during the supervisory visit, which enables more immediate feedback; and expands coverage to include facilities without laboratories. For readability, the approach is referred to herein as OTSS (without the plus sign). The evaluation results are expected to inform decision making by ministries of health, funders, and implementers that are planning to introduce OTSS or, where already implemented, to consolidate and expand the use of the OTSS approach and contribute to the evidence to achieve universal access to high-quality malaria service delivery.

## MATERIALS AND METHODS

### Evaluation objective.

The primary evaluation question was to assess whether current OTSS interventions delivered through the Impact Malaria Project improved the quality of malaria service delivery. The independent evaluation used a mixed-methods design. Qualitative components of the evaluation, including an online survey, in-depth interviews, and focus group discussions, are reported in a separate publication,^13^ whereas this work focuses on a quantitative analysis of OTSS visit records. Further results from the independent evaluation,^14^ along with background information on the IMaD, MalariaCare, and Impact Malaria OTSS projects are available elsewhere.^12^

### Study area and OTSS procedures.

Data from facilities in four countries were included in this study: Cameroon, Ghana, Niger, and Zambia. These four countries were selected purposively to reflect countries with a longer history of OTSS (Ghana and Zambia) and recent adopters (Cameroon and Niger), and with francophone and anglophone settings, and to capture some variability in OTSS implementation strategy and experience. The first iteration of the OTSS program started in 2008 under the U.S. President’s Malaria Initiative (PMI)–funded IMaD Project, which ran until early 2012. The second iteration, the PMI-funded MalariaCare Project, began immediately thereafter and ran until 2017. The PMI-funded Impact Malaria Project, the focus of this evaluation, represents the third iteration of the OTSS program and has benefited from the previous 10 years of networking, capacity building, implementation activities, and operations research. Each successive version of the OTSS program built on the predecessor projects, expanding from a diagnostic-centric improvement approach to encompass malaria case management, overall quality of care, and the use of data for decision making.

During an OTSS visit, supervisors visit a health facility and observe facility staff during patient consultations, completing a standardized checklist during the observation. Separate checklists are used to assess 1) clinical case management of malaria, 2) preparation and interpretation of RDTs, 3) preparation of blood films and reading by light microscopy, and 4) prevention of MIP procedures during routine ANC visits. Additional OTSS checklists are used in some countries but are not part of this evaluation. Checklists are similar among countries, but national malaria control programs adapt and refine template checklists to the local context. Subnational supervisors were generally recruited by regional and district directors based on specified criteria in relation to their technical skills and experience in different malaria service delivery areas. Supervisors complete a training program prior to undertaking OTSS visits, and complete checklists using a mobile-based application that includes skip patterns and calculates summary scores automatically. Supervisors may move in teams to complete multiple checklists concurrently at large facilities or may work independently to complete each checklist consecutively at smaller facilities with fewer staff and lower patient flow. Up to three health worker–client observations are completed for each checklist on each OTSS visit to a facility. When supervisors identify issues, same-day feedback and on-the-job training are provided, and action plans developed to address priority issues. Frequency of OTSS rounds is generally quarterly or biannually, but health facility prioritization differs by country, with some targeting according to geography, and others using previous OTSS performance and/or routine malaria surveillance indicators.

### Data extraction.

Data included in this evaluation were collected during OTSS visits to health facilities in the four target countries under the auspices of Impact Malaria. No additional primary data collection was completed for the purpose of this evaluation. The data available included all indicators collected on OTSS checklists at the individual health worker observation level (up to three health workers observed at each facility and OTSS visit), as well as automatically calculated summary scores for each OTSS checklist domain (clinical, MIP, RDT, and microscopy).

In Cameroon, data comprised three OTSS rounds completed between January 2020 and December 2021. Ghana data included three OTSS rounds from January 2019 to March 2021. Data from Niger included four OTSS rounds from June 2020 to August 2021. Zambia OTSS data covered six OTSS rounds from January 2019 to September 2021.

Indicator definitions across the four countries were checked for consistency of coding prior to pooling data. The OTSS summary checklist scores (clinical, MIP, RDT, microscopy) were converted from a continuous measure (0–100) to a binary indicator of health workers being deemed competent (score ≥90) or not competent (score <90). This threshold was chosen to match the program targets used by Impact Malaria, where a score of ≥90 indicates competence. Because the same health facilities were not necessarily visited in every OTSS round, health facility name was used as a linking variable to calculate the OTSS visit iteration (e.g., first visit, second visit) independent of OTSS round. When individual checklist indicators were missing, it was assumed they were not assessed on the visit and were thus excluded from the data set for the specific outcome indicator.

### Data analysis.

Descriptive analysis of OTSS overall checklist scores over time used spaghetti plots, whereby a line plot is used to display individual health facilities’ scores over each visit iteration, with an overall fitted linear trend to indicate the relationship between OTSS visit iteration and outcome score.

Mixed-effect logistic regression models were generated to assess the association between increasing number of OTSS visits and changes in malaria case management performance as assessed by OTSS checklists. The continuous exposure was the OTSS visit iteration at the facility (i.e., first, second, and third OTSS visit). OTSS visits completed prior to the Impact Malaria Project (e.g., under the MalariaCare or IMaD projects) were not considered in these models (see supplemental information for sensitivity analyses incorporating MalariaCare data). Separate models were generated for each of eight outcome indicators: overall competency (score ≥90%) for the four checklist domains (clinical malaria management, MIP, RDT, microscopy), and four key checklist indicators that reflect critical decision points (if parasitological test is requested appropriately, clinician not prescribing antimalarial drugs to patients with negative malaria test results, supervisor agreement with clinician’s final diagnosis, and whether SP is provided correctly to pregnant women eligible for IPTp) (Table 1).

**Table 1:** Summary of indicators selected for use as primary indicators in models, their checklist source (domain), and outcome definition and coding

Domain checklist	Indicator	Outcome definitions
Clinical case management	Health worker competent in clinical malaria management	1: Competent (score ≥90 on clinical checklist) 0: Not competent (score <90 on clinical checklist)
	Laboratory test was requested appropriately to confirm malaria	1: Test requested appropriately 0: Test not requested appropriately
	Clinician did not prescribe any antimalarial drug to an individual with negative malaria microscopy or RDT result	1: Patient with negative malaria test result not given any antimalarial 0: Antimalarial was given to patient with negative malaria test result
	Supervisor agreed with clinician’s classification of patient as either 1) not malaria, 2) uncomplicated malaria, or 3) severe malaria	1: Supervisor and health worker agreement on classification of patient 0: Supervisor disagrees with health worker classification of patient
Malaria in pregnancy	Health worker competent in prevention of malaria in pregnancy	1: Competent (score ≥90 on malaria-in-pregnancy checklist) 0: Not competent (score <90 on malaria-in-pregnancy checklist)
	Health worker provided three pills of SP to pregnant women eligible for IPTp	1: IPTp was given to eligible women 0: IPTp was not given to eligible women
RDT	Health worker competent in RDT preparation and interpretation	1: Competent (score ≥90 on RDT checklist) 0: Not competent (score <90 on RDT checklist)
Microscopy	Health worker competent in malaria microscopy	1: Competent (score ≥90 on microscopy checklist) 0: Not competent (score <90 on microscopy checklist)

IPTp = intermittent preventive treatment in pregnancy; RDT = rapid diagnostic test; SP = sulfadoxine–pyrimethamine.

Inclusion criteria for the mixed-effect models were 1) the facility received more than one OTSS visit during the Impact Malaria Project (2018–2022) where the specific OTSS checklist was completed and 2) a summary checklist score <90% on the first OTSS visit under Impact Malaria. Logistic regression models included country as a fixed effect, and facility as a random effect to account for nonindependence of observations from the same facilities. These models can be considered a dose—response design, where the dose is the OTSS visit iteration and the response is the checklist-derived score or performance indicator. All analyses were completed using RStudio (version 4.1.0; Posit, Boston, MA) and model fitting was completed with the GLMMadaptive package.^15^

Predicted probabilities and marginal effects of models were used to assess the potential number of OTSS rounds needed in each country to achieve acceptable results across the outcome domains, and to determine where diminishing returns might be relevant to program planners. Predicted probability plots were generated from fitted models for each outcome indicator up to the highest visit iteration observed within the pooled data set; however, this strategy resulted in out-of-range predictions for some countries (e.g., in Ghana where facilities received a maximum of two OTSS visits, but predicted probability plots for some indicators were generated for OTSS visits 3 and 4).

A sensitivity analysis expanded the mixed-effect logistic regression models to include OTSS visits completed in Ghana and Zambia under a previous OTSS implementation mechanism (MalariaCare), incorporating up to 10 total OTSS visits to individual facilities (full methods and results detailed in supplemental information). A second sensitivity analysis modified inclusion criteria for mixed-effect models to allow inclusion of health facilities that met competency thresholds on their first OTSS visit iteration under the Impact Malaria Project. Further sensitivity analyses assessed interactions between country and the eight outcome indicators. All data analysis and reporting followed Strengthening the Reporting of Observational Studies in Epidemiology guidelines.^16^

## RESULTS

Data were available from 46,969 OTSS health worker patient observations completed under the Impact Malaria Project from the clinical checklist (*n =* 13,567), MIP checklist (*n =* 17,758), RDT checklist (*n =* 13,156), and microscopy checklist (*n =* 2,488). A total of 1,893 observations were from Cameroon, 38,080 from Ghana, 1,226 from Niger, and 5,770 from Zambia. Of 5,119 total facilities with any OTSS visits reported under Impact Malaria, 4,635 facilities were excluded from at least one of the final domain-specific data sets because of the availability of checklist data for only one OTSS visit. A total of 4,183 facilities were excluded from all domain-specific data sets because of the receipt of only one OTSS visit. In addition, 542 other facilities were excluded from at least one final domain-specific data set used for logistic regression models because of high performance (overall score ≥90 on checklist) on the first OTSS visit (Table 2).

**Table 2:** Summary of OTSS data from Cameroon, Ghana, Niger, and Zambia: Total OTSS rounds received, reasons for facility exclusion from final domain datasets, and number of facilities retained in each OTSS domain model

Variable	Cameroon	Ghana	Niger	Zambia
Total OTSS rounds under the Impact Malaria Project	3	3	4	6
OTSS rounds under prior project (not included in models)	0	16	0	18
Health facilities receiving an OTSS visit for any of the four checklists under the Impact Malaria Project	445	3,949	103	622
Health facilities excluded from one or more domain models because of the receipt of one OTSS visit for a specific domain	368	3,861	8	398
Health facilities excluded from one or more domain models because of high performance on the first visit for a specific domain	103	124	56	259
Total no. of health facilities remaining in the data set for dose–response models in each domain				
Clinical observation OTSS model	159	109	87	217
Malaria-in-pregnancy observation OTSS model	148	81	85	218
RDT observation OTSS model	42	75	48	95
Microscopy observation OTSS model	5	58	0	0
No. of health facilities receiving the final clinical checklist data set for OTSS visit no.				
1	159	109	87	217
2	159	109	87	217
3	29	0	85	122
4	0	0	61	47
5	0	0	0	6

OTSS = Outreach Training and Supportive Supervision; RDT = rapid diagnostic test.

**Figure 1.**
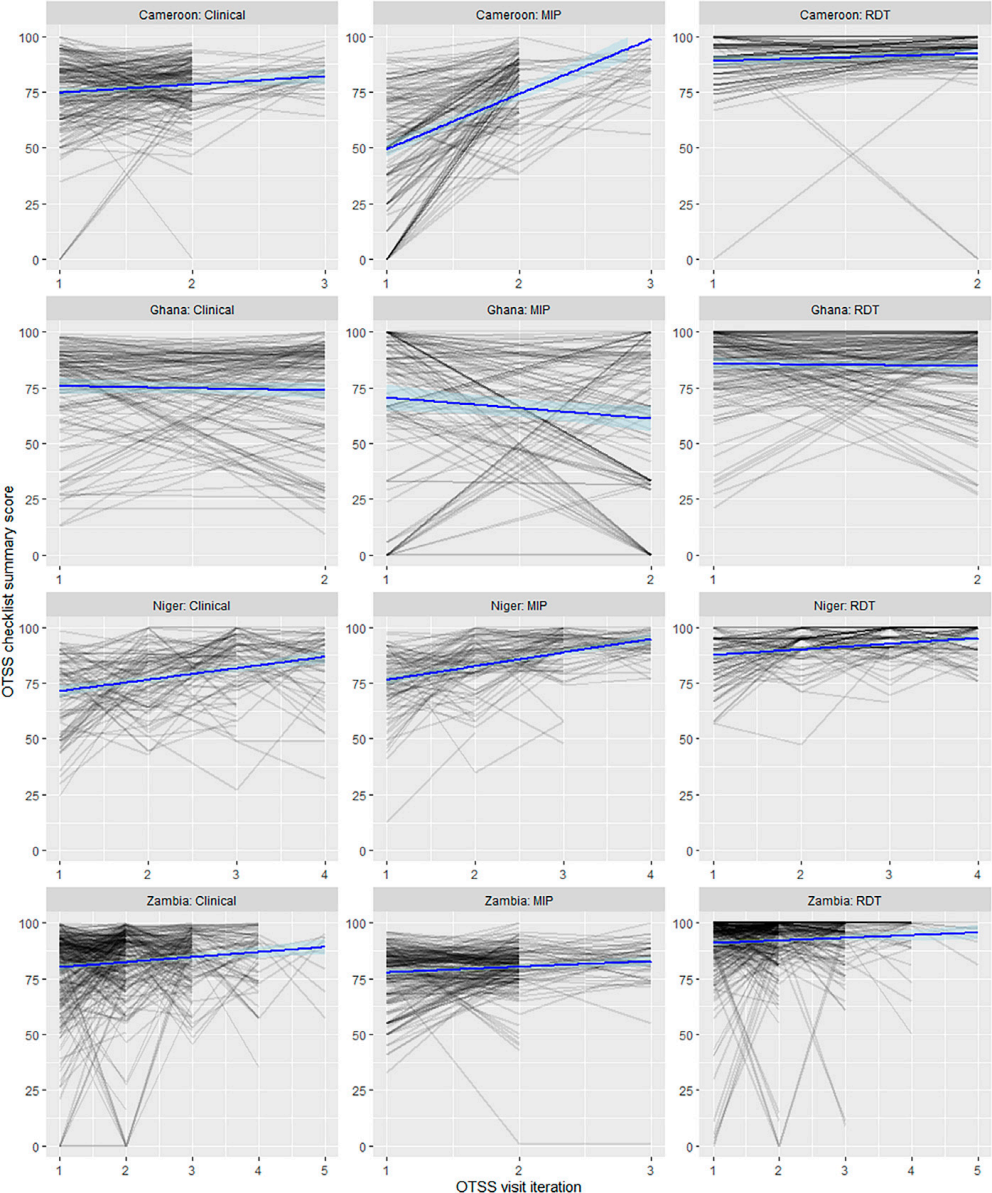
Spaghetti plots describing the performance of individual health facilities over successive OTSS visits (grey lines), and fitted linear trend (blue line). MIP = malaria in pregnancy; OTSS = Outreach Training and Supportive Supervision; RDT = rapid diagnostic test.

Spaghetti plots of all facilities receiving more than one OTSS visit, and the change in checklist score following each successive OTSS visit iteration, are shown in Figure 1. Increasing linear trends are apparent for Cameroon and Niger MIP scores, and Cameroon, Niger, and Zambia clinical scores. Consistent high performance is seen in most facilities for RDT checklist performance.

Mixed-effect logistic regression models using pooled data from the four countries indicate an association between the increasing number of OTSS visits received by a health facility and improving performance. Of the eight outcome indicators assessed in separate models, there was statistical evidence for an effect of increasing OTSS visits on improved performance against seven indicators (Table 3). The only indicator for which no association was found was whether the OTSS supervisor agreed with the clinician’s classification of a patient as no malaria, uncomplicated malaria, or severe malaria (*P* = 0.175). The largest effect estimates were observed for the outcomes of health worker abstaining from providing an antimalarial prescription after negative microscopy or RDT result (odds ratio [OR], 3.80; 95% CI, 2.35–6.16), meaning a more than three times increase in the odds of correct prescribing behavior for each additional OTSS visit made to the facility. Similarly, the odds of administering SP correctly to eligible pregnant women increased by five times for each additional OTSS visit (OR, 5.20; 95% CI, 3.02–8.95). Odds of health workers reaching competency thresholds for MIP increased by more than four times for each additional OTSS visit (OR, 4.62; 95% CI, 3.62–5.88), with similar effect sizes observed for malaria RDT competency (OR, 4.47; 95% CI, 3.62–5.53). A large effect was also seen for malaria microscopy, for which each additional OTSS visit resulted in 10 times the odds of the health worker meeting the competency threshold (OR, 10.73; 95% CI, 4.04–28.52).

**Table 3:** Summary of eight mixed-effect models generated to assess associations between OTSS visit iteration (continuous) received by a facility and key malaria case management outcome indicators*

Primary outcome indicator	Fixed effect	Observations	Health facilities	OR	95% CI	*P* value
Health worker competent in malaria clinical management (overall score ≥90)	OTSS visit iteration, *N*	2,957	572	2.05	1.83–2.30	<0.001
	Zambia (ref.), *n*	1,425	217	1.00	–	–
	Cameroon, *n*	440	159	0.14	0.09–0.21	<0.001
	Ghana, *n*	720	109	0.96	0.72–1.27	0.753
	Niger, *n*	372	87	0.40	0.28–0.57	<0.001
Laboratory test was requested appropriately to confirm malaria	OTSS visit iteration, *N*	2,806	571	1.36	1.12–1.66	0.002
	Zambia (ref.), *n*	1,364	216	1.00	–	–
	Cameroon, *n*	440	159	2.07	1.25–3.44	0.005
	Ghana, *n*	630	109	0.63	0.45–0.88	0.007
	Niger, *n*	372	87	5.07	2.17–11.88	<0.001
Clinician did not prescribe any antimalarial drugs to an individual with negative malaria microscopy or RDT result	OTSS visit iteration, *N*	797	441	3.80	2.35–6.16	<0.001
	Zambia (ref.), *n*	384	176	1.00	–	–
	Cameroon, *n*	224	136	0.02	0.01–0.03	<0.001
	Ghana, *n*	118	76	0.76	0.32–1.81	0.541
	Niger, *n*	71	53	0.76	0.23–2.35	0.611
Supervisor agreed with clinician’s classification of patient as not malaria, uncomplicated, or severe	OTSS visit iteration, *N*	2,465	554	1.15	0.94–1.40	0.175
	Zambia (ref.), *n*	1,358	216	1.00	–	–
	Cameroon, *n*	215	142	0.79	0.45–1.40	0.426
	Ghana, *n*	592	109	0.82	0.52–1.27	0.370
	Niger, *n*	300	87	3.71	1.58–8.72	0.003
Health worker competent in malaria microscopy (overall score ≥90)	OTSS visit iteration, *N*	290	63	10.73	4.04–28.52	<0.001
	Zambia, *n*	0	0	–	–	–
	Cameroon, *n*	10	5	1.73	0.14–22.08	0.662
	Ghana (ref.), *n*	280	58	1.00	–	–
	Niger, *n*	0	0	–	–	–
Health worker competent in malaria RDT (overall score ≥90)	OTSS visit iteration, *N*	1,345	260	4.47	3.62–5.53	<0.001
	Zambia (ref.), *n*	574	95	1.00	–	–
	Cameroon, *n*	89	42	0.88	0.51–1.51	0.639
	Ghana, *n*	456	75	0.82	0.59–1.14	0.243
	Niger, *n*	226	48	0.62	0.40–0.98	0.040
Health worker competent in malaria-in-pregnancy prevention and management (overall score ≥90)	OTSS visit iteration, *N*	1,704	532	4.62	3.62–5.88	<0.001
	Zambia (ref.), *n*	478	218	1.00	–	–
	Cameroon, *n*	401	148	2.25	1.29–3.93	0.004
	Ghana, *n*	507	81	13.37	7.83–22.84	<0.001
	Niger, *n*	318	85	5.35	3.09–9.25	<0.001
Health worker provided three pills of SP to pregnant woman eligible for IPTp	OTSS visit iteration, *N*	1,124	453	5.20	3.02–8.95	<0.001
	Zambia (ref.), *n*	489	218	1.00	–	–
	Cameroon, *n*	119	83	0.28	0.08–1.03	0.056
	Ghana, *n*	386	81	0.35	0.13–0.92	0.034
	Niger, *n*	130	71	0.03	0.01–0.10	<0.001

IPTp = intermittent preventive treatment in pregnancy; OR = odds ratio; OTSS = Outreach Training and Supportive Supervision; ref. = reference; SP = sulfadoxine–pyrimethamine.

* Fixed effects and random effect parameters are not shown.

**Figure 2.**
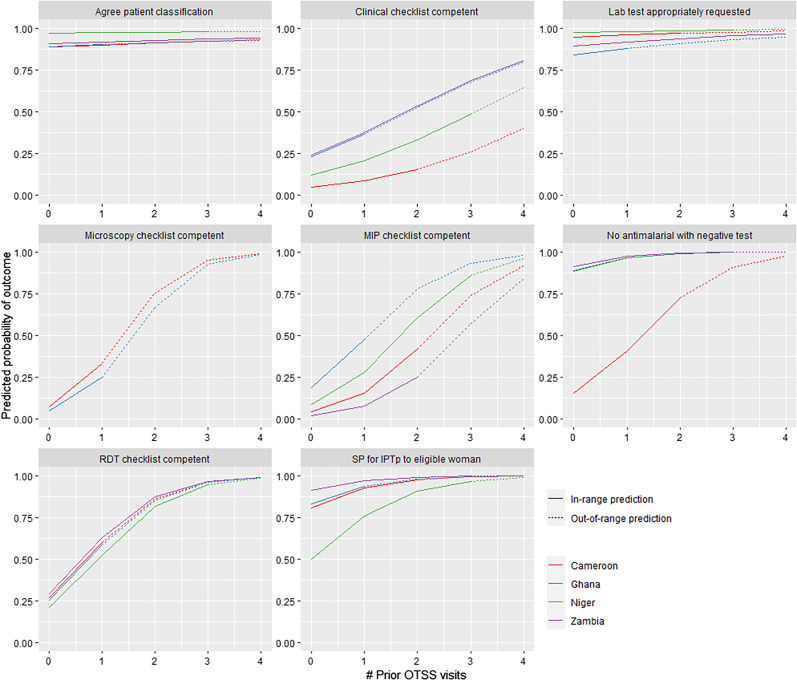
Predicted probability plots illustrating the modeled relationship between increasing number of OTSS visits and predicted probability of successfully achieving the outcome in each country. Out of range predictions (predicting beyond the maximum number of OTSS visits received by facilities for the specific outcome and country) are indicated by dotted lines. IPTp = intermittent preventive treatment in pregnancy; OTSS = Outreach Training and Supportive Supervision; RDT = rapid diagnostic test; SP = sulfadoxine–pyrimethamine.

Predicted probability plots (Figure 2) illustrate that most competency indicators show diminishing returns to greater numbers of OTSS visits on the probability of success scale within the number of visits seen in the OTSS activities to date in the four countries. When the predicted probability of a successful outcome (e.g., competency in RDT or administering SP for IPTp to eligible women) approaches a value of one in predicted probability plots, we consider to have reached saturation. For example, RDT competency appears to reach saturation after facilities have received three prior OTSS visits. Competency in MIP also approaches saturation in Ghana when facilities have received three prior OTSS visits, whereas Cameroon, Niger, and Zambia have not yet reached saturation after the same number of OTSS visits. Other indicators such as agreement on patient classification and requesting laboratory tests appropriately have a high probability of success at baseline (no previous OTSS visits), even after exclusion of facilities from the data set that met overall clinical competency thresholds at their first OTSS visit, indicating that these specific case management performance indicators are met most commonly at visited facilities.

A sensitivity analysis that permitted inclusion of facilities that met competency thresholds on their first OTSS visits found an effect of OTSS for five of the eight outcome indicators: overall clinical competency, forgoing antimalarial prescriptions for patients with a negative malaria test, competency in RDT, competency in MIP prevention, and providing IPTp to eligible women (Supplemental Table S1).

An additional sensitivity analysis of mixed-effect models indicated some evidence for interaction between country and OTSS visit iteration (Supplemental Table S2); however, data set limitations mean there is a low power to investigate fully if effect size differs among countries. Country-specific models for outcomes for which a significant interaction was identified are presented in Supplemental Table S3 and Supplemental Figure S1.

## DISCUSSION

Independent evaluation of OTSS visit data collected in Cameroon, Ghana, Niger, and Zambia during OTSS activities conducted under the Impact Malaria Project demonstrates an impact of increasing number of OTSS visits made to a facility and improving health worker performance as assessed by checklists completed during the OTSS visit. Improvements were seen in both overall summary scores from checklists completed to capture clinical case management of malaria, MIP, RDT and microscopy competency, as well as key indicators such as requesting parasitological tests for eligible patients and abstaining from providing antimalarial drug prescriptions to patients with negative test results. Predicted probability plots indicate diminishing returns in number of OTSS visits received, with four of the eight outcome indicators reaching saturation in all four countries after three or four prior OTSS visits to the facility, and only two indicators where none of the four countries reached saturation after four prior OTSS visits. However, it is an intrinsic characteristic of this type of logistic regression model to show diminishing marginal returns at some point when projecting an increasing number of visits.

Previous work has shown that supportive supervision may lead to improvements in malaria diagnostic performance,^17^^–^^20^ but may also demonstrate diminishing returns over multiple supervisory visits, specifically as it relates to malaria microscopy and RDT performance.^9^^,^^10^ These findings are in line with the current evaluation. Similar to other work, our study also showed small improvements in fever case management practices after supportive supervision activities.^10^^,^^21^ Routine supervision, when used as a sole intervention, as well as an audit with feedback have been associated previously with moderate improvements in health-care worker performance (median, 10.7% and 15.0%, respectively).^22^ In addition, two other studies^23^^,^^24^ found that increasing supervision “dose” was associated with better performance; however, a third review^25^ found that more intensive supervision may not necessarily be more beneficial.

A secondary analysis^22^ of supportive supervision strategies over 36 countries highlighted the role of supervising the supervisors, as well as supervisors engaging health-care workers in problem-solving activities as valuable to enhance the effectiveness of supervision. It is possible that the inclusion of supervisor training as part of the OTSS program activities implemented by Impact Malaria as well as the use of lessons-learned workshops with health-care workers were related to the high effectiveness of OTSS observed in our evaluation.

Our study also differs from analyses of previous malaria OTSS programs by the inclusion of a wider range of primary outcome measures, focusing on specific key steps within the checklist (such as not prescribing antimalarials after receiving negative test results), as well as a binary classification of competency from summary checklist scores, whereas prior evaluations completed linear regressions using the checklist score (0–100) as the outcome measure.^8^^–^^10^ Furthermore, the inclusion of indicators from MIP checklists (overall competency as well as provision of IPTp to eligible women during ANC consultations) has not been reported previously, and suggests that supervision is a valuable tool to support increased IPTp coverage among women attending ANC.

Limitations of our analysis include the outcomes being collected by OTSS supervisors during their visits, with the potential for observer bias as health workers make extra efforts to perform “correctly” while being observed by supervisors; their performance while being observed may not be representative of their usual practices. This may result in bias toward greater performance and could be mitigated by the use of fully independent observations that are not part of routine OTSS activities, or use of other performance measures that do not require direct observation. Nevertheless, the use of repeat observations (during each OTSS round) should mitigate observer bias to some degree.

The relatively small number of third, fourth, and greater OTSS visits to health facilities limits the ability of our analysis to assess fully the extent of diminishing returns with high numbers of OTSS visits. The exclusion of facilities reaching the defined competency thresholds (e.g., score ≥90% on a specific OTSS checklist) on their first OTSS visit may also have exacerbated the effect of regression to the mean for low-performing facilities and may lead to positively biased effect estimates. In addition, the varying strategies used in different countries to select facilities for OTSS visits resulted in some country data sets including relatively few facilities receiving multiple OTSS visits and a large proportion of all facilities excluded as a result of receiving only one OTSS visit during the period of interest. This was particularly true for Ghana, where facilities that had already benefited from OTSS visits were excluded from later rounds, allowing new facilities to participate in OTSS. The small number of observations available for some indicators in certain countries precluded the use of country-specific models, instead pooling data for all four countries to maximize power to assess whether OTSS is linked with improved malaria case management quality. Predicted probability plots illustrate some of the variation by country for each indicator, showing where the largest gains in performance were observed. A further limitation of the available data set for our evaluation is the lack of control or comparison facilities. Because the outcome indicators used for this evaluation are generated by the OTSS visits themselves, it is not possible to include any facilities in this evaluation that did not receive OTSS visits.

Our study focused on OTSS visits completed under the Impact Malaria Project, but both Ghana and Zambia had prior OTSS programs. A sensitivity analysis that combined available data from OTSS completed under the previous PMI-supported mechanism, MalariaCare, with the more recent OTSS data found comparable results to the main analysis, which used more recent OTSS program data (Supplemental Tables S4 and S5). However, possible effect modification by country and lack of sufficient data to investigate this fully potentially limits the generalizability of these results to other country settings.

The use of a dose–response model with the first OTSS visit being a low dose limits the ability to compare high doses of OTSS (many visits) to those facilities receiving a low dose of OTSS. In settings where OTSS visits are prioritized according to previous performance, there are no equivalent facilities receiving multiple visits to compare with facilities with no visits, whereas settings prioritizing wide geographic coverage had few facilities that received a high number of visits. Furthermore, the OTSS checklists are relatively standardized among countries included in our evaluation, limiting our ability to compare relative effect size for different components of OTSS and to make programmatic recommendations about the OTSS components that are most important. Although our evaluation identified successfully the number of prior OTSS visits that result in facilities attaining competency targets, it was not possible (with our study design) to explore questions related to the duration at which high performance can be maintained in the absence of OTSS or the ideal frequency of OTSS visits to a facility.

Last, this evaluation focused only on malaria case management performance as assessed by the observing supervisor and does not explore any patient-level outcomes, demand-side responses, or impact on malaria burden. As a result, it is difficult to explore cost-effectiveness and the importance of OTSS further, particularly in consideration of potential diminishing returns from larger numbers of repeat OTSS visits and the relative impact of other malaria interventions. Although some OTSS data sets do include contextual data such as observed health worker cadre, if they have been supervised previously or received malaria case management training, these data were incomplete, and incorporating these contextual data into models would have reduced substantially the number of observations available for models. Use of data sets external to OTSS to indicators of health worker performance could further strengthen plausibility of the evaluation—for example, through patient exit interviews or health facility survey and/or observations separate to OTSS activities. Attempts were made in the broader independent evaluation to explore the impact of OTSS using routine health management information system (HMIS) data; however, these findings were limited by the lack of suitable indicators of health worker performance within a routine HMIS, relying on imperfect proxy indicators of ratio of total suspected malaria cases to total tested by parasitological test, and a ratio of total confirmed malaria cases to number of patients prescribed ACT.^13^

Our evaluation provides strong evidence that the receipt of multiple OTSS visits to health facilities resulted in measurable improvements in a range of indicators linked to quality case management of patients attending health facilities with suspected malaria, as well quality malaria prevention services received by pregnant women attending ANC. Pending questions include the optimal intensity of OTSS required to maintain proficiency, once acquired. Further evaluation incorporating control facilities would also serve to strengthen further the evidence of OTSS effectiveness. Key considerations for effective OTSS activities remain similar to those discussed in relation to previous OTSS programs,^11^ including the importance of qualified and trained supervisors, prioritization of facilities for visit and follow-up to underperforming facilities, and immediate feedback and action planning to address issues identified during the OTSS visit.

## Supplemental Materials

10.4269/ajtmh.23-0150 Supplemental Materials
