# Supplementary material for: Can Outreach Training and Supportive Supervision Improve Competency in Malaria Service Delivery? An Evaluation in Cameroon, Ghana, Niger, and Zambia
Source: Am J Trop Med Hyg. 2023 Dec 4;110(3 Suppl):10–9. doi: 10.4269/ajtmh.23-0150 (PMC10919235; doi:10.4269/ajtmh.23-0150)
Supplement: Supplemental Materials [file tpmd230150.SD1.pdf]

Supplementary files

Ashton et al. Can outreach, training and supportive supervision improve competency in malaria diagnostic testing and clinical case management: an evaluation in Cameroon, Ghana, Niger, and Zambia

Inclusion of health facilities which met competency thresholds on their first OTSS visit under Impact Malaria in pooled dose-response models – a sensitivity analysis

Method

A sensitivity analysis attempted to assess the impact of decision to remove baseline high performing facilities from dose response models. Baseline high performing facilities are those which scored ≥90% on the specific domain checklist on their first OTSS visit under the Impact Malaria project.

Outcome indicators and mixed effect logistic regression models were otherwise the same as those presented in the main manuscript, whereby facilities receiving only one OTSS visit were excluded, country was retained in models as a fixed effect, and a random effect included for the individual facility to account for non-independence of observations from the same facilities.

Supplemental Table S1: Summary of eight mixed effect models generated to assess association between OTSS visit iteration (continuous) received by a facility and key malaria case management outcome indicators. This sensitivity analysis permitted inclusion of facilities with baseline high performance.

Primary outcome indicator	Fixed effect	N obs	N health facility	OR	95% CI	p-value
Health worker competent in malaria clinical management (overall score ≥90)	**OTSS visit iteration**	4003	756	**1.29**	**1.18, 1.41**	**<0.001**
	Zambia (ref)	2132	323	1.00	-	-
	Cameroon	543	195	0.18	0.13, 0.25	<0.001
	Ghana	927	143	0.68	0.52, 0.90	0.007
	Niger	401	95	0.34	0.24, 0.49	<0.001
Laboratory test was appropriately requested to confirm malaria	**OTSS visit iteration**	**3839**	**755**	**1.15**	**0.98, 1.36**	**0.093**
	Zambia (ref)	2061	322	1.00	-	-
	Cameroon	543	143	1.60	1.00, 2.56	0.048
	Ghana	834	95	0.61	0.44, 0.86	0.004
	Niger	401	322	4.18	1.88, 9.30	<0.001
Clinician did not prescribe any antimalarial drug to an individual with negative malaria microscopy or RDT result	**OTSS visit iteration**	**1098**	**587**	**4.15**	**2.72, 6.32**	**<0.001**
	Zambia (ref)	589	263	1.00	-	-
	Cameroon	277	166	0.02	0.01, 0.03	<0.001
	Ghana	156	100	0.92	0.43, 1.99	0.842
	Niger	76	58	0.81	0.26, 2.47	0.708
Supervisor agreed with clinician's classification of patient as not malaria / uncomplicated / severe	**OTSS visit iteration**	**3440**	**734**	**1.03**	**0.87, 1.23**	**0.707**
	Zambia (ref)	2058	322	1.00	-	-
	Cameroon	264	174	0.60	0.36, 1.01	0.057
	Ghana	794	143	0.73	0.48, 1.10	0.127
	Niger	324	95	2.99	1.33, 6.72	0.008

Primary outcome indicator	Fixed effect	N obs	N health facility	OR	95% CI	p-value
Health worker competent in malaria microscopy (overall score ≥90)	**OTSS visit iteration**	**394**	**83**	**1.12**	**0.67, 1.88**	**0.666**
	Zambia	0	0	-	-	-
	Cameroon	14	7	1.16	0.18, 7.52	0.878
	Ghana (ref)	380	76	1.00	-	-
	Niger	0	0	-	-	-
Health worker competent in malaria RDT (overall score ≥90)	**OTSS visit iteration**	**3659**	**683**	**1.33**	**1.20, 1.48**	**<0.001**
	Zambia (ref)	2008	317	1.00	-	-
	Cameroon	246	112	0.95	0.63, 1.42	0.806
	Ghana	957	158	0.41	0.31, 0.54	<0.001
	Niger	448	96	0.64	0.45, 0.92	0.015
Health worker competent in malaria in pregnancy prevention & management (overall score ≥90)	**OTSS visit iteration**	**2130**	**622**	**1.96**	**1.67, 2.31**	**<0.001**
	Zambia (ref)	545	246	1.00	-	-
	Cameroon	408	151	1.07	0.68, 1.69	0.771
	Ghana	813	129	8.50	5.82, 12.42	<0.001
	Niger	364	96	4.91	3.21, 7.51	<0.001
Health worker provided 3 pills of SP to pregnant woman eligible for IPTp	**OTSS visit iteration**	**1466**	**542**	**3.32**	**2.14, 5.16**	**<0.001**
	Zambia (ref)	556	246	1.00	-	-
	Cameroon	121	85	0.33	0.09, 1.16	0.085
	Ghana	643	129	0.58	0.24, 1.40	0.223
	Niger	146	82	0.03	0.01, 0.09	<0.001

Stratum-specific mixed effect models for association between increasing OTSS visit iterations and malaria case management indicators

Method

Pooled models were tested with interaction terms between country and OTSS visit iteration for each of the defined outcomes. A likelihood ratio test was used to test the null hypothesis that there was no interaction between country and OTSS visit iteration in each case management outcome model. Where there was evidence for an interaction, country-specific models were generated for further description of the effect of OTSS on malaria case management in each country.

Results

Evidence for interaction between country and OTSS visit iteration is indicated for six of the outcomes assessed. Microscopy competence could not be assessed since the model with interaction term did not successfully converge.

In stratum-specific models, an effect of increasing OTSS rounds on health worker competence in malaria clinical management and malaria in pregnancy management were seen in all four countries, although with varying effect size. Effects on successful patient classification, and on withholding antimalarials from patients with negative tests were only found in Cameroon. Effect of increasing OTSS rounds on RDT competency was found in Ghana, Niger, and Zambia but not in Cameroon. Improvements in IPTp provision with increasing OTSS rounds were found in Ghana and Niger.

The primary limitation of the country-specific models is the small amount of data for some outcomes, limiting the ability to fully assess the impact of OTSS on outcomes within these country-specific models.

Supplemental Table S2: Likelihood ratio test results comparing models with and without interaction term between country and OTSS visit iteration

Primary outcome indicator	Likelihood ratio test statistic	Degrees of freedom	p
Health worker competent in malaria clinical management (overall score ≥90)	18.93	3	0.0003
Laboratory test was appropriately requested to confirm malaria	3.46	3	0.3263
Clinician did not prescribe any antimalarial drug to an individual with negative malaria microscopy or RDT result	57.56	3	<0.0001
Supervisor agreed with clinician's classification of patient as not malaria / uncomplicated / severe	42.32	3	<0.0001
Health worker competent in malaria microscopy (overall score ≥90)	N/A[1]		
Health worker competent in malaria RDT (overall score ≥90)	39.27	3	<0.0001
Health worker competent in malaria in pregnancy prevention & management (overall score ≥90)	12.38	3	0.0062
Health worker provided 3 pills of SP to pregnant woman eligible for IPTp	47.23	3	<0.0001

[1]*Model with interaction term did not converge*

Supplemental Table S3: Country-specific model results for the six outcomes where interaction between country and OTSS visit iteration was indicated

Primary outcome indicator	Country	N obs	N hf	OR	Effect estimate 95% CI	p-value
Health worker competent in malaria clinical management (overall score ≥90)	Zambia	**1425**	**217**	**1.75**	**1.53, 2.00**	**<0.001**
	Cameroon	**440**	**159**	**17.71**	**2.55, 123.27**	**0.004**
	Ghana	**720**	**109**	**3.02**	**2.08, 4.38**	**<0.001**
	Niger	**372**	**87**	**2.35**	**1.80, 3.05**	**<0.001**
Clinician did not prescribe any antimalarial drug to an individual with negative malaria microscopy or RDT result	Zambia	384	176	0.84	0.46, 1.53	0.565
	Cameroon	**224**	**136**	**42.77**	**5.38, 339.86**	**<0.001**
	Ghana	118	76	3.36	0.02, 983.81	0.676
	Niger	71	53	2.05	0.55, 7.62	0.282
Supervisor agreed with clinician's classification of patient as not malaria / uncomplicated / severe	Zambia	1358	216	1.00	0.81, 1.25	0.968
	Cameroon	**215**	**142**	**87.86**	**5.31, 1453**	**0.002**
	Ghana	592	109	0.93	0.52, 1.66	0.795
	Niger	300	87	0.92	0.45, 1.87	0.819
Health worker competent in malaria RDT (overall score ≥90)	Zambia	**574**	**95**	**4.29**	**3.14, 5.86**	**<0.001**
	Cameroon	89	42	1254.93	0.23, $6.7e^{6}$	0.104
	Ghana	**456**	**75**	**5.16**	**3.32, 8.00**	**<0.001**
	Niger	**226**	**48**	**3.03**	**2.11, 4.34**	**<0.001**
Health worker competent in malaria in pregnancy prevention & management (overall score ≥90)	Zambia	**478**	**218**	**4.93**	**2.36, 10.31**	**<0.001**
	Cameroon	**401**	**148**	**34.16**	**6.23, 187.27**	**<0.001**
	Ghana	**507**	**81**	**2.58**	**1.70, 3.90**	**<0.001**
	Niger	**318**	**85**	**5.56**	**3.59, 8.61**	**<0.001**
Health worker provided 3 pills of SP to pregnant woman eligible for IPTp	Zambia	489	218	1.34	0.71, 2.56	0.369
	Cameroon	119	83	1.02	0.11, 9.33	0.985
	Ghana	**386**	**81**	**5784.38**	**149.08, $2.2e^{5}$**	**<0.001**
	Niger	**130**	**71**	**3.13**	**1.49, 6.59**	**0.003**

Supplemental Figure S1: Forest plot displaying estimated effect of increasing OTSS visit iteration on the six outcomes where there was evidence for interaction between country and OTSS iteration. For each outcome, the effect estimate from country-specific models (supplementary table 5) and corresponding effect estimate from pooled model (Table 3) are presented.

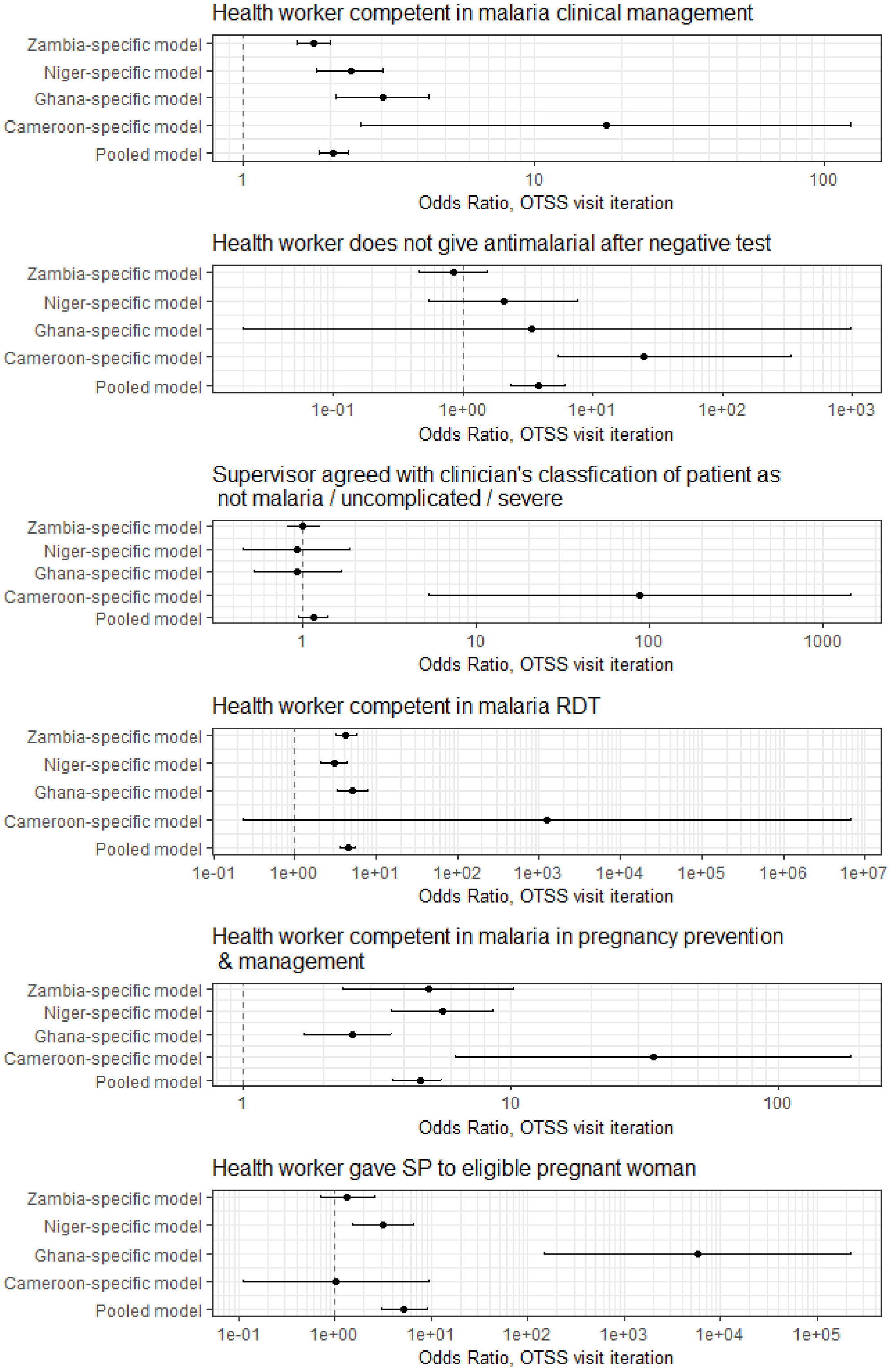

Inclusion of OTSS visits completed under MalariaCare from 2014-2018 in Ghana and Zambia in pooled dose-response models – a sensitivity analysis

Method

A sensitivity analysis attempted to further investigate associations between increasing OTSS visits (as a continuous exposure) and summary OTSS checklist scores combining visits under MalariaCare and Impact Malaria. This analysis was intended to capture the longer history of OTSS in Ghana and Zambia, where facilities may have received more OTSS rounds.

MalariaCare data was available summarized by facility-round (not facility and health worker observation), necessitating aggregation of Impact Malaria data to the same level. Combining the Impact Malaria and MalariaCare datasets assumed that health facility names remained consistent between the two projects, although it should be noted that this likely underestimates the extent of repeat visits across the two project eras, since some facilities may have existed under different, un-linkable names in both datasets.

Health facilities which had received only one OTSS visit during the Impact Malaria project were excluded from the logistic regression models dataset. In addition, health facilities which were high performing on their first visit were excluded from the dataset: high performing was defined as scoring ≥90 on the relevant checklist (e.g. for outcome indicators on the clinical checklist this was defined an overall clinical checklist score ≥90), as these facilities could not be measurably improved by further OTSS intervention. Logistic regression models included country as a fixed effect and had a random effect for the individual facility to account for non-independence of observations from the same facilities.

Models were prepared for the summary 'competence' indicators for the overall clinical, RDT and microscopy checklists, where competence was defined as scoring ≥90% on the checklist. Malaria in pregnancy scores were not available for the MalariaCare project.

Supplemental Table S4: Summary of OTSS data available from Cameroon, Ghana, Niger, and Zambia for sensitivity analysis combining OTSS visits from MalariaCare and Impact Malaria projects

	**Cameroon**	**Ghana**	**Niger**	**Zambia**
Total OTSS rounds under MalariaCare project (2014-2018)	0	16	0	18
Total OTSS rounds under Impact Malaria project (2019-2021)	3	3	4	6
Health facilities excluded: only one OTSS visit	368	3873	8	520
Health facilities excluded: high performance on first visit	103	1087	56	333
Total health facility remaining in dataset for dose response models				
Clinical observation OTSS models	158	898	87	296
RDT observation OTSS models	42	754	48	156
Microscopy observation OTSS models	5	58	0	6
Number of health facilities receiving Nth OTSS visit, final clinical checklist dataset				
1st	158	898	87	296
2nd	158	898	87	296
3rd	29	504	85	194
4th	0	189	61	95
5th	0	33	0	43
6th	0	4	0	25
7th	0	0	0	17

	Cameroon	Ghana	Niger	Zambia
8th	0	0	0	6
9th	0	0	0	1
10th	0	0	0	0

Results

Effect estimates for models combining data from OTSS visits conducted under MalariaCare and Impact Malaria in the four focus countries were similar to the effect estimates reported for Impact Malaria data only. It was not possible to fit a model to the combined data for the microscopy competency outcome.

While the combined dataset includes OTSS visits from higher visit iterations (up to a 6th visit in Ghana and up to a 9th visit in Zambia), data in the combined models were summarized to single outcome for each health facility visit, rather than separate scores for each observed health worker. As a result, power in the combined model may have been reduced compared to the main model using data from the most recent OTSS activities only.

Supplemental Table S5: Summary of mixed effect models generated to assess association between OTSS visit iteration and key malaria case management outcome indicators, using a combination of OTSS data from MalariaCare and Impact Malaria projects

Impact Malaria & MalariaCare combined OTSS scores	**Denominators**		**OTSS effect estimate (combined data)**			**Effect estimate, Impact Malaria data only**
	**Obs**	**Health facility**	**OR**	**95% CI**	**p-value**	
Health worker competent in malaria clinical management (overall score ≥90)	4166	1439	2.05	1.89, 2.23	<0.001	2.05 (1.83, 2.30)
Health worker competent in malaria microscopy (overall score ≥90)	143	69	N/A[1]			10.73 (4.04, 28.52)
Health worker competent in malaria RDT (overall score ≥90)	3004	1001	3.59	3.25, 3.98	<0.001	4.47 (3.62, 5.53)

[1]*Model did not converge, no output available*
